# Case Report: Uromodulin-associated autosomal dominant tubulointerstitial kidney disease: case analysis and literature review

**DOI:** 10.3389/fneph.2026.1931661

**Published:** 2026-10-29

**Authors:** Linlin Zheng, Hui Gao, Xiangkai Zheng, Xing Zhang, Xiaona Wei

**Affiliations:** 1 Department of Nephrology (Second Ward), Hebei Provincial Hospital of Chinese Medicine, Shijiazhuang, China; 2 Hebei Key Laboratory of Turbidity Toxin Syndrome, Shijiazhuang, China; 3 Hebei Technology Innovation Center of Traditional Chinese Medicine (TCM) Spleen and Kidney Diseases, Shijiazhuang, China

**Keywords:** ADTKD-UMOD, case report, chronic kidney disease, hyperuricemia, uromodulin

## Abstract

Autosomal dominant tubulointerstitial kidney disease caused by *UMOD* variants (ADTKD-UMOD) is a rare hereditary disorder characterized by early-onset hyperuricemia, gout, and progressive kidney dysfunction. Diagnosis is established by genetic testing. We report the case of a 25-year-old man who presented with elevated serum creatinine (Scr) and uric acid (UA) levels. His father and elder sister had chronic kidney disease of unknown etiology, suggesting an autosomal dominant inheritance pattern. A comprehensive evaluation excluded secondary causes of hyperuricemia and kidney dysfunction. Genetic testing identified a novel heterozygous missense variant, *UMOD* (NM_003361.4): c.274T>A (p.Cys92Ser), in exon 3 of the gene. The same variant was also detected in affected relatives. In silico analyses predicted a deleterious effect, and the variant was classified as likely pathogenic according to the ACMG/AMP criteria (PM5_Moderate, PP3_Moderate, PM2_Supporting). During longitudinal follow-up with febuxostat treatment, serum urate levels decreased, while progressive deterioration of renal function was observed during the observational period. By analogy to well-characterized pathogenic cysteine-altering *UMOD* variants, we hypothesize that p.Cys92Ser may promote uromodulin misfolding and endoplasmic reticulum retention and thereby contribute to kidney injury; this mechanistic inference remains to be verified by direct functional experiments for this novel variant. This report expands the mutational spectrum of ADTKD-UMOD and underscores the importance of early genetic testing in at-risk families, as well as standardized long-term follow-up and management for this progressive disorder.

## 1 Introduction

Autosomal dominant tubulointerstitial kidney disease (ADTKD) comprises a group of hereditary kidney disorders characterized by chronic tubulointerstitial injury and progressive loss of renal function. The uromodulin-associated subtype (ADTKD-UMOD), caused by pathogenic variants in the *UMOD* gene on chromosome 16, is the most common form. Epidemiological data from a single UK center reported a prevalence of 9 per million in the general population and approximately 1% among adults with chronic kidney disease (CKD) stages 3-5 (1, 2). Large-scale exome sequencing studies have further shown that UMOD variants account for about 3% of CKD cases with a confirmed monogenic cause, indicating that ADTKD-UMOD is rare but clinically relevant (3). ADTKD-UMOD is inherited in an autosomal dominant manner with near-complete penetrance, and affected individuals typically develop manifestations during adolescence or early adulthood. Hallmark features include early-onset hyperuricemia, gout, and progressive kidney dysfunction, which may ultimately lead to end-stage kidney disease (ESKD).

Uromodulin is produced by epithelial cells of the thick ascending limb of the loop of Henle and plays an important role in urinary homeostasis, including regulation of salt-water handling, defense against urinary tract infection, prevention of kidney stone formation, and immune modulation. Most pathogenic *UMOD* variants are located in exons 3 and 4 and can cause protein misfolding, endoplasmic reticulum stress, and subsequent tubulointerstitial fibrosis. Because ADTKD-UMOD lacks specific histopathological features, misdiagnosis is common, and genetic testing remains the diagnostic gold standard. Although advances in sequencing technologies have improved diagnostic yield, reports describing novel variants and their associated clinical phenotypes remain limited.

In this study, we report a case of ADTKD-UMOD associated with a novel heterozygous missense variant, c.274T>A (p.Cys92Ser), in exon 3 of the *UMOD* gene. Based on family history, clinical manifestations, laboratory findings, and genetic testing, a definitive diagnosis was established, and the pathogenicity of the variant was systematically evaluated. In addition, the proband and affected relatives underwent longitudinal follow-up to assess changes in renal function and serum uric acid levels. We draw mechanistic hypotheses by reference to other cysteine-disrupting *UMOD* variants, recognizing that functional validation for p.Cys92Ser is currently absent. This report expands the known mutational spectrum of ADTKD-UMOD and provides evidence to support early diagnosis, cascade genetic screening in at-risk families, and improved long-term clinical management.

## 2 Case presentation

The patient was a 25-year-old man who presented to our hospital for evaluation of impaired renal function. One year earlier, a routine health examination had shown elevated serum creatinine (Scr, 173 μmol/L; reference range, 57-97 μmol/L) and uric acid (UA, 618 μmol/L; reference range, 202-428 μmol/L), with an estimated glomerular filtration rate (eGFR) of 46 mL/min/1.73m² (adult reference range, 90–120 mL/min/1.73m²). Urinalysis at that time showed a specific gravity of 1.020 (reference range, 1.003-1.03), urine pH of 5.0 (reference range, 4.5-8.0), and was negative for protein and occult blood. However, these abnormal findings were overlooked. He had been prescribed febuxostat 40 mg daily, but adherence was inconsistent. After admission, repeat laboratory tests confirmed persistently elevated renal indices. The patient denied any significant past medical history, long-term medication use, smoking, alcohol consumption, or drug allergies. Previous checkups, including routine urinalysis, had reportedly been unremarkable.

Family history was notable for kidney dysfunction in first-degree relatives. The patient’s father was first found to have elevated Scr, BUN, and UA 12 years ago at the age of 42 years; he is now 54 years old and receiving peritoneal dialysis. The patient’s older sister was found to have elevated Scr, BUN, and UA during prenatal examination of her first pregnancy 9 years ago at age 26. Because febuxostat is contraindicated during pregnancy, urate-lowering therapy was not initiated. She subsequently underwent lactation and a second pregnancy, and urate-lowering treatment remained deferred. After systematic laboratory assessment in May 2024, hyperuricemia was confirmed, and regular oral febuxostat was commenced thereafter. Nevertheless, her Scr has continued to increase progressively. She is now 35 years old, with an Scr level of 324 μmol/L and an eGFR of 15 mL/min/1.73m². Clinical data for other family members were not available.

The patient underwent comprehensive ancillary testing. Urinary N-acetyl-β-D-glucosaminidase, parathyroid hormone (PTH), thyroid function, and an autoimmune panel—including antineutrophil cytoplasmic antibody (ANCA), myeloperoxidase-ANCA (MPO-ANCA), proteinase 3-ANCA (PR3-ANCA), perinuclear ANCA (p-ANCA), antinuclear antibody (ANA), anti-double-stranded DNA antibody (anti-dsDNA), anti-phospholipase A2 receptor antibody (anti-PLA2R), and anti-glomerular basement membrane antibody (anti-GBM)—were all within normal limits. Immunoglobulin quantification, hepatitis B serology, and serum protein electrophoresis were also unremarkable. Renal color Doppler ultrasonography showed bilaterally normal kidney size and contour with smooth capsules, mildly increased parenchymal echogenicity, and blurred corticomedullary differentiation. No collecting system dilatation was observed. The bilateral renal arteries had a normal course and caliber, with normal ostial wall thickness, good luminal patency, and normal intraluminal flow signals. Flow velocity and spectral waveforms were normal, with no abnormal perfusion signals detected (**Figure 1**).

**Figure 1:**
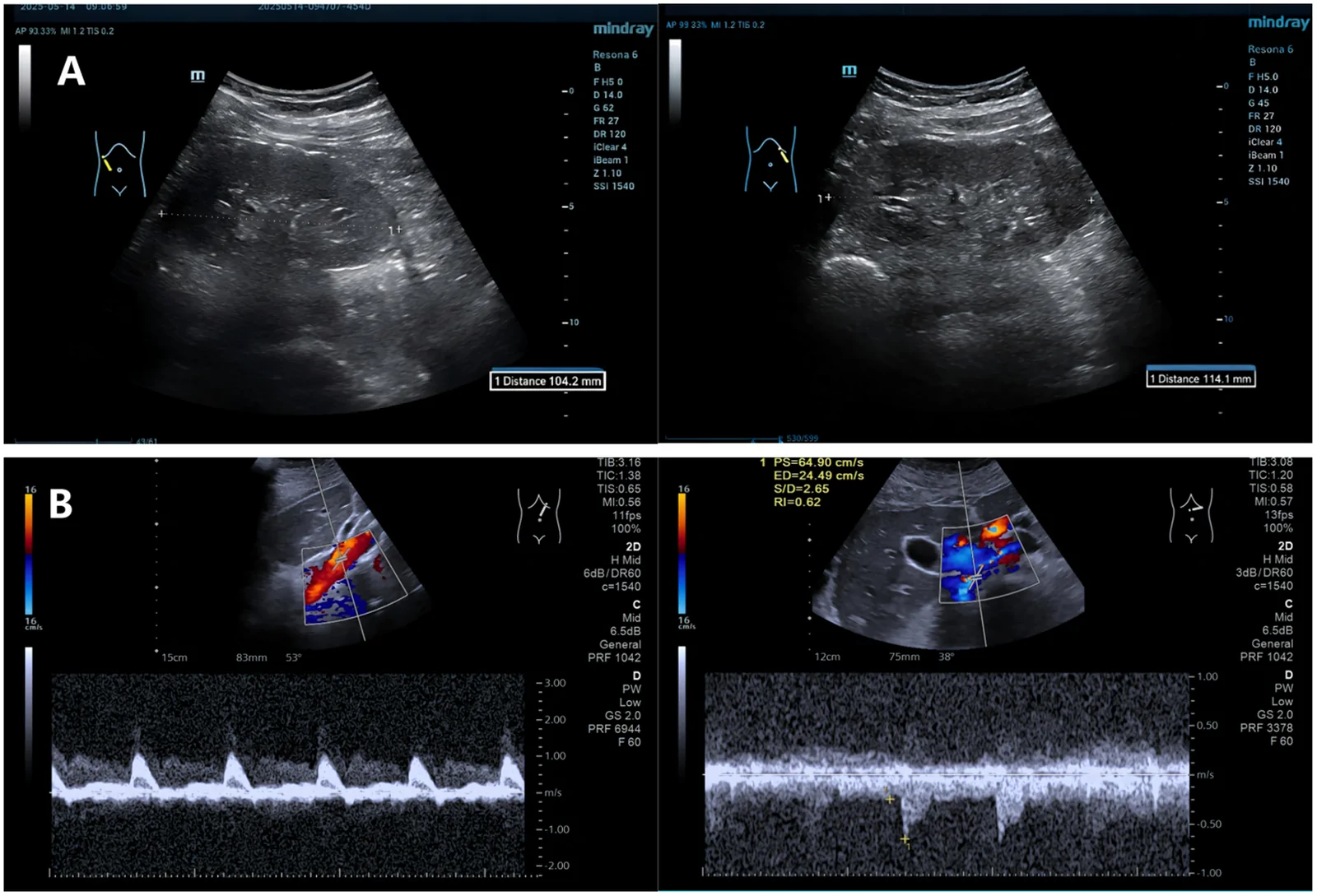
Ultrasonographic images of the patient’s kidneys and renal arteries. **(A)** Ultrasonography of bilateral kidneys; **(B)** ultrasonography of bilateral renal arteries.

CKD of unknown etiology was identified in three family members spanning two consecutive generations, strongly suggesting an autosomal dominant inheritance pattern. Accordingly, following genetic counseling, genetic testing was performed in the patient, his father, and his elder sister. Genomic DNA was isolated from peripheral-blood leukocytes of the proband and available family members. Whole-exome sequencing combined with mitochondrial-genome sequencing was performed. The assay covered all coding exons and adjacent canonical splice-site regions of human nuclear genes as well as the complete mitochondrial genome. The mean coverage depth for whole-exome targets was 100×, with >99.9 % of target bases achieving ≥20× coverage; the mitochondrial genome was sequenced at a mean depth of 3000×. Reads were aligned against human reference assembly GRCh37/hg19. Single-nucleotide variants and small indels were called and functionally annotated. Variant filtering criteria included: removal of variants with population allele frequency >0.01 % in gnomAD_exome, ExAC and 1000 Genomes; retention of variants within coding or splice-site regions; prioritization of variants matching clinical phenotypes of chronic kidney disease, hyperuricemia and renal failure. The heterozygous *UMOD* c.274T>A variant detected by whole-exome sequencing was validated by Sanger sequencing in the proband, his father and elder sister (**Figure 2**). Copy-number-variation (CNV) analysis derived from exome data identified no clinically significant CNVs. Mitochondrial-variant analysis revealed no variants with definite clinical pathogenicity.

**Figure 2:**
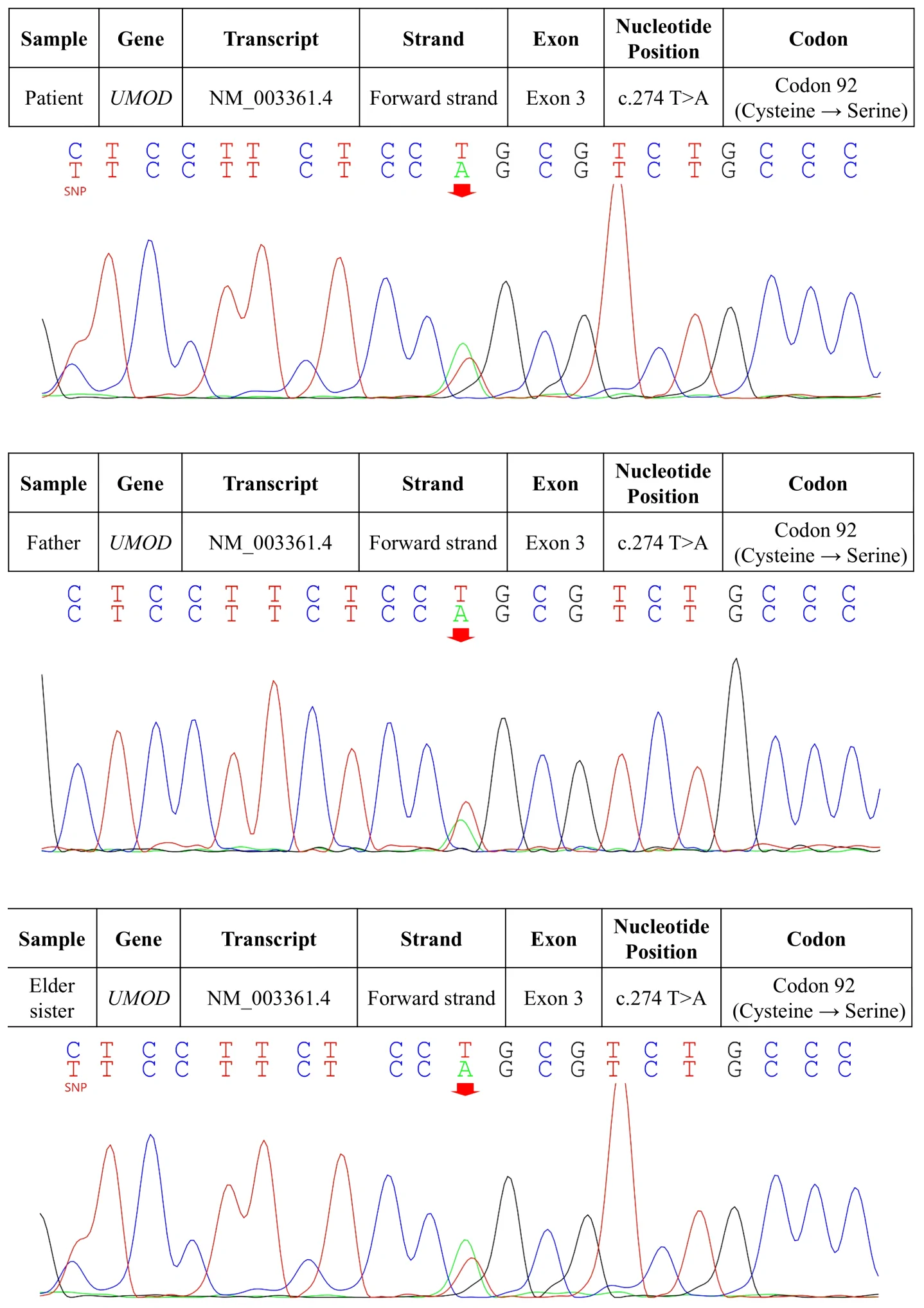
Sanger sequencing chromatograms of the proband, his father and elder sister. Sequencing was performed on the forward strand of *UMOD* reference transcript NM_003361.4. The heterozygous variant c.274T>A alters codon 92 within exon 3, resulting in amino-acid substitution p.Cys92Ser (genomic coordinate chr16:g.20360349T>A). The variant nucleotide position is indicated by red arrow. All three affected family members harbor this heterozygous variant.

The results revealed that the same heterozygous variant in exon 3 of the *UMOD* gene was identified in all three individuals: a thymine-to-adenine substitution at nucleotide 274 (c.274T>A), resulting in a cysteine-to-serine substitution at amino acid position 92 (p.Cys92Ser). According to the American College of Medical Genetics and Genomics (ACMG-AMP 2015) criteria, this variant was classified as likely pathogenic, supported by PM5_Moderate, PP3_Moderate and PM2_Supporting evidence (**Supplementary Table S1**). The cysteine-92 residue lies within the well-known cysteine-rich mutational hotspot of UMOD exon 3; this feature is discussed as gene-specific contextual evidence but was not counted as separate ACMG evidence (PM1) to avoid signal overlap with PM5 and PP3. Complete co-segregation between the heterozygous variant *UMOD* (NM_003361.4): c.274T>A (p.Cys92Ser) and ADTKD-UMOD phenotype was observed across two generations in this pedigree; this pedigree finding is presented as a clinical supportive observation and was not applied as ACMG evidence code PP1 for variant classification, given the limited number of affected family members. The genetic findings were consistent with the patients’ clinical phenotypes and family history (**Figure 2**). The patient was therefore diagnosed with ADTKD-UMOD.

Variant interpretation followed the ACMG-AMP 2015 guidelines. Databases used for variant annotation included HGMD Professional (release 30 June 2025), OMIM (release 31 August 2025), and HPO (release 31 August 2025). The reference transcript for *UMOD* is NM_003361.4, corresponding protein accession NP_003352.2, genome assembly GRCh37/hg19. The variant is described with HGVS nomenclature: genomic coordinate chr16:g.20360349T>A; cDNA c.274T>A; protein p.Cys92Ser. Two informative meioses were observed within this two-generation pedigree. Given that ≥4 informative meioses are required for PP1-Moderate evidence according to ACMG-AMP recommendations, co-segregation was treated as a clinical supportive observation and was not used for variant-scoring as PP1 evidence. A pedigree diagram showing age, genotype, phenotype, renal outcome and tested/untested status for each relative is provided in **Figure 3**.

**Figure 3:**
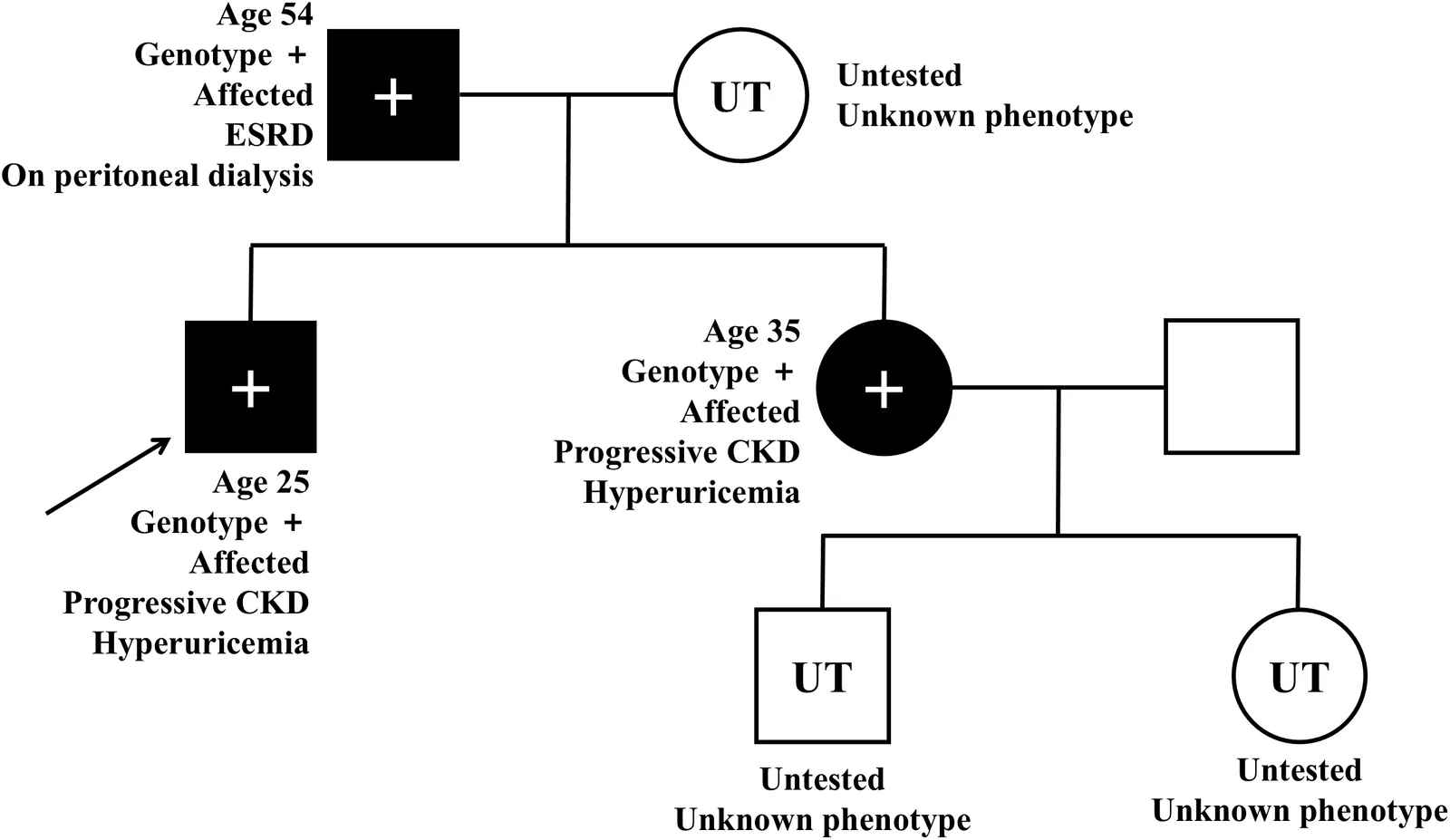
Pedigree of the family with ADTKD-UMOD caused by *UMOD* c.274T>A (p.Cys92Ser). Pedigree showing age, genotype, clinical phenotype, renal outcome and genetic-testing status of each family member. Filled symbols: affected individuals; open symbols (without UT label): unaffected individuals; open symbols labelled UT, untested individuals with unknown phenotype. +: carries *UMOD* c.274T>A variant; −: wild-type; UT, untested. Three affected individuals (proband, father, elder sister) are heterozygous carriers. The father has progressed to ESRD treated with peritoneal dialysis; the proband and elder sister exhibit progressive CKD with hyperuricemia. Two informative meioses are present in this two-generation pedigree.

The novel heterozygous *UMOD* variant c.274T>A (p.Cys92Ser) identified in this pedigree was deposited in the Leiden Open Variation Database (LOVD). Only de-identified aggregated family data without personally identifiable information were used for submission. The LOVD Individual ID is 00487373 (Lab ID: UMOD-2026-01).

Physical examination findings of the proband: The patient was normotensive on initial admission, with serial blood-pressure readings ranging from 118-132/72–84 mmHg. No edema of the lower extremities, no rash, and no joint swelling or tenderness were noted. No other significant physical abnormalities were identified. Comorbidities: The proband had no documented comorbid diseases. Repeated 24-hour urinary total protein was <0.15 g/24 h, consistent with minimal-range proteinuria. For the father: Serial blood-pressure values ranged from 125-146/76–90 mmHg. No significant comorbidities other than end-stage kidney disease. Quantitative urinary protein assessment was not available after initiation of peritoneal dialysis. For the elder sister: Serial blood-pressure readings were 122-140/74–86 mmHg. No significant comorbidities. Repeated 24-hour urinary total protein was <0.15 g/24 h, showing minimal proteinuria.

Treatment information: All three affected family members received oral febuxostat 40 mg once-daily for hyperuricemia. The treatment duration spanned from 2023 to 2025. Medication adherence was inconsistent for the proband, with occasional self-discontinuation; his father and elder sister maintained relatively regular intake. No obvious febuxostat-related adverse reactions (hepatic injury, skin rash, gastrointestinal intolerance) were reported. No gout flares were recorded during follow-up. No dose adjustment of febuxostat was performed during follow-up. The proband had intermittent self-initiated treatment interruptions associated with poor adherence, while his father and elder sister maintained consistent daily oral intake. No patient reported drug-related subjective discomfort. The target serum urate was set below 360 μmol/L. Concomitant medications included antihypertensive agents for blood-pressure control; no other nephrotoxic agents were applied during observation.

Longitudinal monitoring of UA, Scr, and eGFR was performed in the three affected family members, and the changes over time are shown in **Figures 4** and **5**. Under febuxostat treatment, serum urate decreased compared with pre-treatment levels in all three individuals, though urate levels fluctuated substantially and were repeatedly above the pre-defined therapeutic target (< 360 μmol/L). The proband exhibited an obvious serum-urate elevation on March 24, 2024, which coincided with self-reported poor medication adherence. The elder sister’s serum urate gradually stabilized after standardized febuxostat administration. During the observational period, progressive deterioration of renal function was observed in all three subjects, reflected by rising serum creatinine and declining estimated glomerular filtration rate (eGFR). The father’s serum creatinine rose to 1614 μmol/L in June 2025, at which point peritoneal dialysis was commenced. Serum-creatinine levels subsequently decreased and fluctuated due to dialytic solute clearance; this reduction does not represent stabilization or improvement of native intrinsic kidney function. Scr and complete longitudinal eGFR trajectories for all three affected family members are presented in **Figures 4**, **5** and **Supplementary Table S2**. eGFR was calculated using the CKD-EPI 2009 creatinine (CKD-EPI-Cr) equation with units of mL/min/1.73 m². The full computational formula is provided in the **Supplementary Material**. Creatinine-based eGFR was not calculated or reported for samples collected after initiation of maintenance dialysis, as this equation is not valid for dialysis-dependent patients.

**Figure 4:**
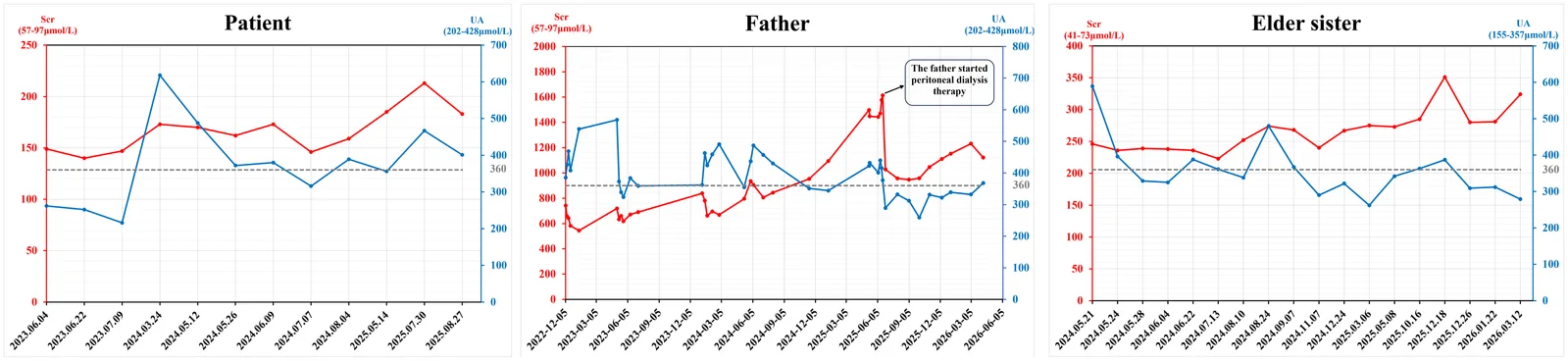
Dynamic changes in UA and Scr levels of the proband, his father and elder sister (tests performed in our hospital). The reference range for adult eGFR is 90–120 mL/min/1.73 m².

**Figure 5:**
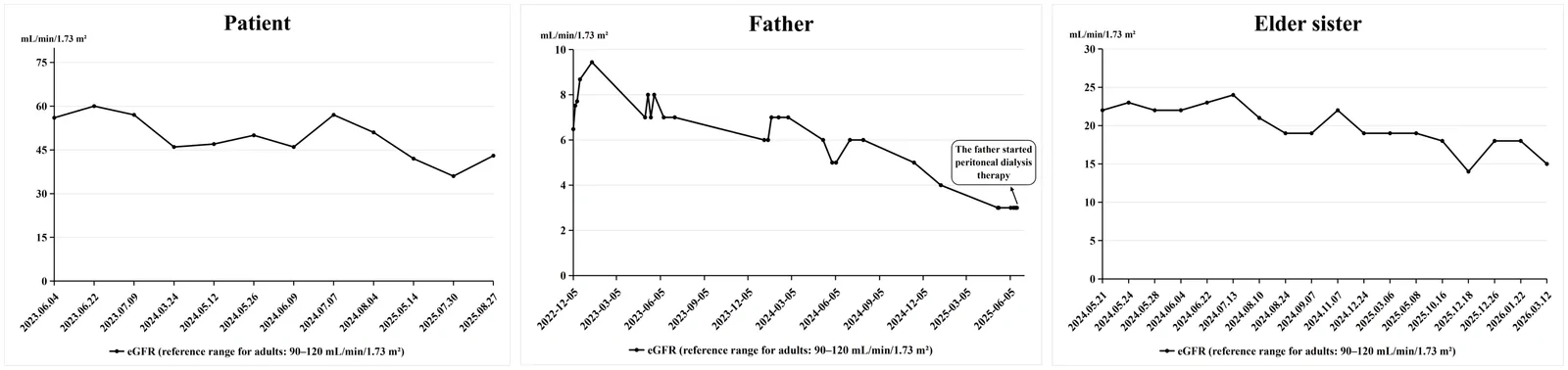
Longitudinal eGFR changes in three affected family members (proband, his father and elder sister).

Note: Left Y-axis for Scr (μmol/L; reference range: 57-97 μmol/L for males, 41-73 μmol/L for females). Right Y-axis for UA (μmol/L; reference range: 202-428 μmol/L for males, 155-357 μmol/L for females). The horizontal dashed line indicates the serum urate therapeutic target of < 360 μmol/L.

Diagnostic reasoning: We initially considered secondary causes of CKD including hypertensive nephropathy, glomerulonephritis, tubulointerstitial nephritis, obstructive nephropathy, and toxic-drug-related renal injury, all of which were excluded through laboratory testing, immunological work-up and renal ultrasound. The two-generation family history with multiple affected relatives strongly suggested an autosomal-dominant hereditary kidney disorder. Clinical features including early-onset hyperuricemia, minimal proteinuria, bland urine sediment, and non-specific renal ultrasound findings were highly suggestive of ADTKD. Subsequent germline genetic testing identified the novel heterozygous UMOD c.274T>A (p.Cys92Ser) variant, which co-segregated with disease phenotype in affected family members, establishing the final diagnosis of ADTKD-UMOD.

Patient-reported outcomes and patient perspective: During follow-up, the proband denied persistent fatigue, bone-joint pain or obvious gastrointestinal discomfort under febuxostat therapy. He expressed concern regarding progressive decline in renal function and future risk of end-stage kidney disease. He hoped for regular laboratory surveillance and early warning of renal deterioration and supported cascade genetic screening for at-risk young family members. The father and elder sister reported no drug-related complaints; they were anxious about long-term renal prognosis and accepted regular monitoring and disease-modifying supportive management. Written informed consent was obtained to report these patient-perspective clinical contents.

## 3 Discussion

### 3.1 ADTKD-UMOD

ADTKD-UMOD is an autosomal dominant tubulointerstitial kidney disease caused by pathogenic variants in the *UMOD* gene. It is considered one of the most common monogenic kidney diseases. This disorder was reported as the most frequent non-polycystic inherited kidney disease in a single-center cohort study; however, conventional whole-exome sequencing cannot reliably detect *MUC1* pathogenic variants, leading to systematic under-ascertainment of ADTKD-MUC1 and potential bias in comparative frequency estimates. Because renal biopsy often lacks characteristic histopathological findings, the disease is frequently underdiagnosed or misdiagnosed in clinical practice. Typical clinical features include (4–7) (1): an autosomal dominant pattern with affected individuals across multiple generations (2); insidious decline in renal function, with an annual reduction in eGFR, normal or mildly reduced kidney size on ultrasonography, minimal or absent proteinuria (<1 g/d), and bland urinary sediment (3); non-specific histology characterized predominantly by tubular atrophy and interstitial fibrosis, without pathognomonic findings.

ADTKD-UMOD, early-onset hyperuricemia and gout are hallmark manifestations, with gout attacks often occurring before 30 years of age. The prevalence of gout and gout-free survival differ significantly from those in other ADTKD subtypes, such as ADTKD-MUC1 (1, 2, 8). Some affected individuals also experience recurrent urinary tract infections or pyelonephritis (2, 9, 10). However, clinically significant edema is typically absent, and although hypertension may develop, it is usually not a prominent feature in the early stages. ADTKD-UMOD commonly begins in adolescence or young adulthood and presents as slowly progressive chronic kidney dysfunction. CKD typically develops in early adulthood, and a proportion of patients progress to ESKD between 40 and 60 years of age (1, 2, 8, 9).

### 3.2 Uromodulin

Uromodulin (Tamm-Horsfall protein) is a high-molecular-weight glycoprotein produced by epithelial cells of the thick ascending limb of Henle’s loop. Synthesized as a GPI-anchored membrane precursor, it undergoes proteolytic cleavage and post-translational processing before release into the tubular lumen, where it assembles into high-molecular-weight filamentous polymers. As the most abundant endogenous protein in normal human urine, uromodulin maintains tubular and urinary homeostasis, participating in water-electrolyte balance, anti-infective defense against uropathogens, and inhibition of nephrolithiasis. It modulates renal immune responses and electrolyte transport via multiple effector pathways including NKCC2, TLR2/TLR4, and TRPV5 (11–13). In disease settings, misfolded mutant uromodulin can disturb these physiological cascades and trigger tubulointerstitial injury.

#### 3.2.1 Inhibition of nephrolithiasis and defense against urinary tract infection

Uromodulin exerts dual protective effects by preventing stone formation and reducing susceptibility to infection. Studies in *UMOD* knockout mice have shown that loss of uromodulin results in renal papillary calcification and ureteral stone formation, supporting its role in inhibiting calcium crystal aggregation and adhesion (10). In addition, glycosylated domains of uromodulin can bind uropathogens and form a molecular trapping network that enhances urinary immune defense. The increased susceptibility to urinary tract infection in *UMOD*-deficient models further supports this protective role (10, 14, 15).

#### 3.2.2 Electrolyte balance and blood pressure regulation

Uromodulin contributes to blood pressure regulation by modulating NKCC2 activity in the thick ascending limb and promoting NCC phosphorylation in the distal tubule, both of which are critical for sodium reabsorption and extracellular volume homeostasis (11, 16, 17). *UMOD* knockout or defective uromodulin maturation has been associated with increased urinary sodium excretion, impaired adaptation to salt loading, altered salt sensitivity, transporter dysfunction, endoplasmic reticulum stress, and tubular injury (11, 16). Moreover, genome-wide association studies (GWAS) have linked *UMOD* variants and uromodulin expression levels to hypertension, salt sensitivity, and CKD risk, further supporting its role in cardiorenal regulation (10, 14, 18, 19).

#### 3.2.3 Bidirectional immunomodulation in renal injury and repair

Uromodulin has context-dependent effects in kidney injury and repair. It can bind immune mediators such as IgG, C1q, TNF-α, and IL-1β and interact with immune cells through TLR4-related pathways (10, 20–22). Experimental studies suggest that *UMOD* deficiency reduces phagocyte activity, aggravates acute kidney injury (AKI), and impairs M2 macrophage-mediated repair, whereas uromodulin supplementation may partially reverse these effects (21). Conversely, interstitially localized uromodulin may function as a damage-associated molecular pattern (DAMP), inducing TNF-α release and amplifying inflammation (10, 23). *UMOD* deletion has also been linked to more severe post-AKI fibrosis, while exogenous uromodulin or its EGF-like domain may promote EGFR degradation, attenuate fibrosis, and facilitate tissue repair (24).

Patients with ADTKD-UMOD commonly exhibit hyperuricemia, tubulointerstitial injury, and progressive CKD, and some may also develop nephrolithiasis. These findings support the role of uromodulin in urinary homeostasis, urate handling, and prevention of stone formation. GWAS further indicate that *UMOD* variants are associated with CKD progression, blood pressure dysregulation, and cardiovascular risk, underscoring the importance of uromodulin in both renal and systemic homeostasis (14, 18, 19, 25).

### 3.3 Genetic characteristics

ADTKD-UMOD is an autosomal dominant disorder with near-complete penetrance, provided affected individuals live long enough to manifest symptoms. However, the disorder shows significant variability in age of onset and disease progression, both within and across families (1, 5, 26, 27). The UMOD gene, located on chromosome 16 (16p12.3), is the most common causative gene for ADTKD, with over 90% of pathogenic variants identified in exons 3 and 4. These variants are primarily heterozygous missense variants clustered in the N-terminal cysteine-rich region (spanning amino acids 28-289). Many of these variants directly affect cysteine residues, disrupting intramolecular disulfide bond formation. This disruption compromises the three-dimensional folding and structural integrity of the uromodulin protein. Such molecular dysregulation is consistently observed in multigenerational pedigrees and is central to the pathogenesis of ADTKD-UMOD (9, 28–30).

ADTKD-UMOD is caused by gain-of-function variants in *UMOD*, in which misfolded mutant uromodulin exerts cytotoxic effects and triggers renal injury. The absence of characteristic renal pathological changes in *Umod* global knockout mice supports the notion that simple uromodulin deficiency is insufficient to cause the disease. *UMOD* variants exhibit distinct allele-specific effects, with different variants causing varying degrees of uromodulin aggregation and renal injury, as demonstrated in gene knock-in mouse models. Deletion of the wild-type allele further aggravates protein aggregation and endoplasmic reticulum stress, highlighting the dose-dependent effects of toxic protein accumulation (30). These findings are consistent with the absence of characteristic renal histological abnormalities in *Umod* global knockout mice, further supporting the conclusion that ADTKD-UMOD is a gain-of-function disorder in which renal injury is mediated by the accumulation of misfolded, toxic uromodulin. Clinically, highly penetrant variants are typically associated with the classic hyperuricemic form of ADTKD-UMOD. In contrast, variants with intermediate effects, such as p.Thr62Pro, are more common in the general population and are associated with milder phenotypes, including moderately reduced urinary uromodulin levels and mild CKD. Nevertheless, even these relatively mild variants may substantially increase the risk of kidney disease, underscoring their clinical relevance (31).

In the present case, the *UMOD* (NM_003361.4): c.274T>A (p.Cys92Ser) variant was identified as a previously unreported missense variant and was classified as likely pathogenic. This variant was not identified in the public databases or published literature searched in this study. Multiple in silico analyses consistently supported its potential deleterious effect. The REVEL score for the UMOD c.274T>A variant was 0.869, which is well above the commonly used threshold of 0.5 and supports a deleterious effect. SpliceAI did not predict a significant impact on splicing, whereas other in silico prediction tools consistently indicated that the variant was damaging. Specifically, SIFT predicted that the variant was deleterious, suggesting that the amino acid substitution may impair protein function; PolyPhen-2 classified it as probably damaging, indicating a potential effect on protein structure or function; and MutationTaster predicted it to be disease-causing.

According to the 2015 ACMG-AMP 2015 standards (32), and guidelines together with ClinGen SVI recommendations for UMOD-related variants, the variant fulfills the moderate-strength ACMG criterion PM5. This criterion is supported by the published pathogenic variant p.Cys92Gly affecting the identical amino-acid residue, whose independent pathogenicity in ADTKD-UMOD patients has been confirmed in primary literature (1). Consistent deleterious in-silico predictions provide PP3_Moderate evidence, and absence in population databases provides PM2_Supporting evidence. Given the substantial signal overlap between PM1 (hot-spot cysteine residue), PM5 and PP3 for cysteine-disrupting UMOD variants, PM1 was not included as a separate scoring criterion per ClinGen-SVI expert panel guidance. Nonetheless, it is well-established that cysteine-disrupting missense variants within this UMOD cysteine-rich domain are highly likely to be pathogenic, which serves as important gene-specific contextual evidence supporting our final likely-pathogenic classification.

The c.274T>A (p.Cys92Ser) described here alters the Cys92 residue. A previously reported pathogenic variant affecting this identical residue is p.Cys92Gly (1). Both variants substitute the conserved cysteine at position 92 and are predicted to disrupt local disulfide-bond formation within exon 3-encoded cysteine-rich domain. Clinically, patients carrying p.Cys92Gly also manifested early-onset hyperuricemia, progressive chronic kidney disease consistent with ADTKD-UMOD, which shows phenotypic similarity to our pedigree. Variants altering adjacent cysteine residues (for example Cys83, Cys95 within the same hotspot domain) are also reported in ADTKD-UMOD; most of these cysteine-missense variants produce a typical ADTKD-UMOD phenotype with early hyperuricemia and progressive renal decline, though inter-individual and intra-familial phenotypic variability is frequently observed. While ACMG-AMP 2015 criteria support classification of p.Cys92Ser as likely pathogenic, direct functional data for p.Cys92Ser is still lacking, and the mechanistic inference of uromodulin misfolding and ER retention is extrapolated from these analogous cysteine-altering *UMOD* variants rather than proven experimentally for this specific variant.

Nevertheless, we acknowledge an important limitation: we did not perform *in-vitro* functional experiments to directly demonstrate uromodulin misfolding and endoplasmic reticulum stress triggered by p.Cys92Ser. Functional assays would further consolidate its pathogenic mechanism, which represents a limitation of the present report. Further functional studies are warranted to clarify the molecular mechanisms by which the c.274T>A (p.Cys92Ser) variant contributes to renal injury.

In addition, archived biological specimens for retrospective measurement of serum/urinary uromodulin and fractional excretion of uric acid (FEUA) are no longer available for this family, so these supporting laboratory markers could not be assessed in our present study.

### 3.4 Hyperuricemia in ADTKD-UMOD

Approximately 70% of UA is eliminated through the kidneys, In CKD, a decline in the glomerular filtration rate (GFR) reduces urate filtration and thereby contributes to underexcretion-type hyperuricemia. In addition, CKD may promote renal urate retention by altering proximal tubular urate handling, including enhanced urate reabsorption mediated by transporters such as URAT1 and GLUT9. These changes may be influenced by several factors, including activation of the renin–angiotensin-aldosterone system (RAAS), volume depletion, and insulin resistance. Collectively, these mechanisms exacerbate hyperuricemia in patients with CKD (33–35).

ADTKD-UMOD represents a distinct model of early-onset hyperuricemia that develops despite relatively preserved GFR. Pathogenic variants in *UMOD* can impair NKCC2 expression or function in the thick ascending limb, leading to reduced sodium reabsorption and mild volume depletion (1, 36, 37). In response, compensatory sodium and fluid reabsorption in the proximal tubule may enhance urate reabsorption through increased activity or expression of urate transporters such as URAT1 and GLUT9. This process reduces the fractional excretion of uric acid (FEUA) and contributes to underexcretion-type hyperuricemia (1, 36, 38, 39). This mechanism is supported by clinical findings of elevated serum urate, reduced FEUA, and early-onset gout, which may occur during childhood or adolescence (1, 39). Experimental studies in relevant Umod-deficient mouse models have also provided support for altered proximal tubular urate handling, including increased URAT1 expression and hyperuricemia (36–38). Accordingly, ADTKD-UMOD commonly presents as hereditary tubulointerstitial kidney disease accompanied by early-onset hyperuricemia and, in some patients, recurrent gout.

Febuxostat, a xanthine oxidase inhibitor, lowers serum urate levels by suppressing urate production. In the present study, longitudinal clinical observations demonstrated that febuxostat was effective in correcting hyperuricemia in the affected patients. However, no clear benefit in slowing the decline in renal function was observed. This finding may be explained by the distinct mechanisms underlying urate handling and renal injury in ADTKD-UMOD. Renal urate reabsorption and transport occur predominantly in the proximal tubule and involve several transporters, including URAT1, GLUT9, and OAT4. Although febuxostat effectively reduces urate biosynthesis by inhibiting xanthine oxidase, it does not correct the primary molecular defect in ADTKD-UMOD—namely, the intracellular accumulation of misfolded mutant uromodulin. This accumulation induces endoplasmic reticulum stress and promotes tubulointerstitial injury. In our small family-based observational series, serum urate decreased under febuxostat treatment, but progressive renal dysfunction persisted across the observation period. Given the limitations of this uncontrolled case series (variable adherence, heterogeneous CKD stages, irregular sampling, one subject receiving dialysis), we cannot draw definitive conclusions regarding whether febuxostat exerts nephroprotective or disease-modifying effects in ADTKD-UMOD. Larger prospective studies are required to clarify this clinical question.

### 3.5 Management

The prevalence of ADTKD is likely higher than currently estimated because of substantial clinical underdiagnosis. Initial screening can be guided by a family history consistent with autosomal dominant inheritance, bland urinary findings, and subtype-specific clinical features. The *UMOD* subtype commonly presents with early-onset hyperuricemia and gout, *whereas* the *REN* subtype is characterized by childhood hypotension, anemia, hyperkalemia, and metabolic acidosis. The *HNF1B* subtype is frequently associated with congenital anomalies of the kidney and urinary tract (CAKUT) and impaired glucose metabolism, while the *MUC1* subtype typically presents as isolated CKD (5, 27, 40). Importantly, no pathognomonic histological finding is shared across all subtypes. Therefore, as recommended by KDIGO, genetic testing remains the gold standard for definitive diagnosis, rather than renal biopsy as a first-line diagnostic tool (6, 26, 41, 42).

After ADTKD-UMOD is confirmed by genetic testing, management focuses on slowing CKD progression, treating hyperuricemia and gout, delaying progression to ESKD, and providing family screening and genetic counseling. Because no curative therapy is currently available, care relies on close longitudinal monitoring of eGFR, blood pressure, proteinuria, electrolytes, and serum urate levels (1, 5, 42). ESKD typically develops between 50 and 54 years of age, with an average eGFR decline of approximately 1–3 mL/min/1.73m² per year (1, 29). Management should follow standard CKD principles, including strict blood pressure control, cardiovascular risk reduction, and avoidance of nephrotoxic exposures such as NSAIDs and contrast agents. Renin–angiotensin system blockers (e.g., losartan) may be preferred because of their uricosuric effects (1, 5). Because early-onset hyperuricemia and gout are hallmark features (with a median gout-free survival of approximately 30 years), targeted management of these complications is important. When hyperuricemia or gout is present, urate-lowering therapy should be initiated and dose-adjusted according to renal function. Patients should also be advised to maintain a healthy lifestyle, ensure adequate hydration, follow a low-purine diet, and avoid diuretics and alcohol where possible.

ADTKD-UMOD follows an autosomal dominant inheritance pattern; therefore, first-degree relatives of affected individuals have a 50% risk of carrying the familial pathogenic variant. Genetic counseling and cascade testing are essential for early diagnosis, risk stratification, and informed decision-making (5, 41, 42). Potential related living kidney donors should undergo genetic testing, and only non-carriers should be considered eligible donors. Kidney transplantation remains the definitive treatment for kidney failure in ADTKD-UMOD and is considered curative, with no recurrence in the allograft reported to date; thus, early transplant referral should be considered as eGFR declines (1, 5, 42). Biomarkers such as urinary and serum uromodulin are promising tools that may aid diagnosis and disease monitoring (1, 43). Although current treatment is mainly supportive, ongoing research into targeted therapies—including approaches to correct uromodulin misfolding, reduce endoplasmic reticulum stress, and limit fibrosis—offers hope for future disease-modifying interventions (2, 5, 42). An integrated multidisciplinary strategy combining long-term CKD care, urate/gout control, family surveillance, and timely transplant planning is critical for optimizing outcomes.

## 4 Conclusion

In conclusion, this case supports a pathogenic association between a novel *UMOD* variant and ADTKD-UMOD, highlighting the central role of genetic testing and cascade family screening in the diagnosis and management of hereditary kidney disease. Based on comprehensive evaluation of clinical manifestations, laboratory findings, genetic testing, pedigree data and ACMG-AMP variant assessment, the diagnosis of ADTKD-UMOD was established. Our findings provide supportive evidence for the likely-pathogenic nature of this novel *UMOD* variant and add to the current understanding of ADTKD-UMOD. ADTKD-UMOD is an autosomal dominant kidney disease characterized by early-onset hyperuricemia, gout, and progressive renal dysfunction. Genetic testing remains essential for definitive diagnosis. By inference from other known cysteine-disrupting pathogenic *UMOD* variants, this novel variant is hypothesized to promote uromodulin misfolding and endoplasmic reticulum retention, thereby contributing to tubulointerstitial injury; this mechanistic hypothesis awaits direct functional experimental validation for p.Cys92Ser. Long-term observational follow-up demonstrated that serum urate was controlled under febuxostat treatment, while progressive renal impairment was observed in affected family members. Clinical management should emphasize family screening, regular laboratory surveillance, and strict avoidance of nephrotoxic agents. In advanced disease, timely referral for kidney transplantation is critical to optimize outcomes.

## Data Availability

The original contributions presented in the study are included in the article/**Supplementary Material**. Further inquiries can be directed to the corresponding author.
