# Supplementary material for: Case Report: Uromodulin-associated autosomal dominant tubulointerstitial kidney disease: case analysis and literature review
Source: Front Nephrol. 2026 Oct 29;6:1931661. doi: 10.3389/fneph.2026.1931661 (PMC13754732; doi:10.3389/fneph.2026.1931661)
Supplement: Supplementary file 1 [file Table1.docx]

**Supplementary information**

Title: Uromodulin-Associated Autosomal Dominant Tubulointerstitial Kidney Disease: Case Analysis and Literature Review

**Table S1. Full ACMG‑AMP 2015 variant‑interpretation evidence matrix for *UMOD* NM_003361.4: c.274T>A (p.Cys92Ser) (genomic coordinate chr16:g.20360349T>A, GRCh37/hg19)**

| **Evidence code** | **Evidence strength** | **Evidence description** |
| --- | --- | --- |
| PM5 | Moderate | A different pathogenic missense substitution affecting the identical amino‑acid residue (p.Cys92Gly) has been reported for ADTKD‑UMOD. |
| PP3 | Moderate | Multiple computational predictors consistently support deleterious effect: REVEL = 0.869; SIFT = Deleterious; PolyPhen‑2 = Probably damaging; MutationTaster =  Disease‑causing. |
| PM2 | Supporting | Variant is absent in gnomAD_exome, ExAC, 1000 Genomes and East‑Asian population datasets; zero allele count in large general‑population controls. |
| Final Classification | — | Likely Pathogenic (PM1_Moderate + PM5_Moderate + PP3_Moderate + PM2_Supporting) |
| Additional clinical observation (not used for ACMG scoring) | Supporting | Complete co‑segregation of heterozygous variant with ADTKD‑UMOD phenotype observed in a two‑generation pedigree (three affected carriers). |

Footnote: Genome build: GRCh37/hg19; Genomic coordinate: chr16:g.20360349T>A; Reference transcript: *UMOD* NM_003361.4; Protein accession: NP_003352.2. Two informative meioses were observed in this two‑generation pedigree. PP1 evidence was not applied for variant classification because ≥4 informative meioses are required for PP1‑Moderate strength per ACMG‑AMP 2015 standards.

**Table S2 Raw Scr, UA, and eGFR Data of the Patient, His Father, and Elder Sister**

| **Patient** | | | |
| --- | --- | --- | --- |
| Time | Scr (57-97μmol/L) | UA (202-428μmol/L) | eGFR (90-120 mL/min/1.73 m²) |
| 2023.06.04 | 149 | 262 | 56 |
| 2023.06.22 | 140 | 252 | 60 |
| 2023.07.09 | 147 | 216 | 57 |
| 2024.03.24 | 173 | 618 | 46 |
| 2024.05.12 | 170 | 488 | 47 |
| 2024.05.26 | 162 | 372 | 50 |
| 2024.06.09 | 173 | 380 | 46 |
| 2024.07.07 | 146 | 316 | 57 |
| 2024.08.04 | 159 | 389 | 51 |
| 2025.05.14 | 185 | 356 | 42 |
| 2025.07.30 | 213 | 467 | 36 |
| 2025.08.27 | 183 | 401 | 43 |

| **Father** | | | |
| --- | --- | --- | --- |
| **Time** | **Scr (57-97μmol/L)** | **UA (202-428μmol/L)** | eGFR (90-120 mL/min/1.73 m²) |
| 2022-12-05 | 742 | 385 | 6.48 |
| 2022-12-09 | 657 | 426 | 7.52 |
| 2022-12-13 | 643 | 469 | 7.71 |
| 2022-12-19 | 583 | 408 | 8.68 |
| 2023-01-13 | 544 | 539 | 9.44 |
| 2023-05-04 | 719 | 568 | 7 |
| 2023-05-10 | 633 | 373 | 8 |
| 2023-05-16 | 660 | 339 | 7 |
| 2023-05-23 | 618 | 324 | 8 |
| 2023-06-12 | 671 | 384 | 7 |
| 2023-07-05 | 690 | 359 | 7 |
| 2024-01-08 | 839 | 362 | 6 |
| 2024-01-16 | 782 | 463 | 6 |
| 2024-01-23 | 662 | 424 | 7 |
| 2024-02-07 | 696 | 459 | 7 |
| 2024-02-27 | 669 | 491 | 7 |
| 2024-05-10 | 796 | 355 | 6 |
| 2024-05-29 | 935 | 437 | 5 |
| 2024-07-05 | 806 | 457 | 6 |
| 2024-06-06 | 906 | 487 | 5 |
| 2024-08-02 | 844 | 430 | 6 |
| 2024-11-16 | 953 | 351 | 5 |
| 2025-01-11 | 1094 | 344 | 4 |
| 2025-05-10 | 1498 | 422 | 3 |
| 2025-05-12 | 1448 | 432 | 3 |
| 2025-06-06 | 1443 | 401 | 3 |
| 2025-06-12 | 1471 | 440 | 3 |
| 2025-06-16 | 1577 | 415 | 3 |
| 2025-06-19 | 1614 | 377 | 3 |
| 2025-06-27 | 1028 | 289 | - |
| 2025-08-01 | 957 | 332 | - |
| 2025-09-04 | 947 | 312 | - |
| 2025-10-04 | 958 | 259 | - |
| 2025-11-03 | 1047 | 331 | - |
| 2025-12-09 | 1111 | 322 | - |
| 2026-01-04 | 1152 | 339 | - |
| 2026-03-04 | 1232 | 332 | - |
| 2026-04-09 | 1122 | 368 | - |

| **Elder sister** | | | |
| --- | --- | --- | --- |
| **Time** | **Scr (41-73μmol/L)** | **UA (155-357μmol/L)** | eGFR (90-120 mL/min/1.73 m²) |
| 2024.05.21 | 246 | 589 | 22 |
| 2024.05.24 | 236 | 396 | 23 |
| 2024.05.28 | 239 | 329 | 22 |
| 2024.06.04 | 238 | 325 | 22 |
| 2024.06.22 | 236 | 388 | 23 |
| 2024.07.13 | 223 | 361 | 24 |
| 2024.08.1 | 252 | 338 | 21 |
| 2024.08.24 | 274 | 480 | 19 |
| 2024.09.07 | 268 | 367 | 19 |
| 2024.11.07 | 240 | 290 | 22 |
| 2024.12.24 | 267 | 322 | 19 |
| 2025.03.06 | 275 | 262 | 19 |
| 2025.05.08 | 273 | 342 | 19 |
| 2025.10.16 | 285 | 363 | 18 |
| 2025.12.18 | 351 | 387 | 14 |
| 2025.12.26 | 280 | 309 | 18 |
| 2026.01.22 | 281 | 312 | 18 |
| 2026.03.12 | 324 | 279 | 15 |

Note: eGFR calculation formula

The CKD‑EPI 2009 creatinine equation:

For males:

If Scr ≤ 0.9 mg/dl : eGFR=141×(Scr/0.9)^-0.411^×0.993^age^

If Scr ＞ 0.9 mg/dl : eGFR=141×(Scr/0.9)^-1.209^×0.993^age^

For females:

If Scr ≤ 0.7 mg/dl : eGFR=144×(Scr/0.7)^-0.329^×0.993^age^

If Scr ＞ 0.7 mg/dl : eGFR=144×(Scr/0.7)^-1.209^×0.993^age^

where 144 = 141 × 1.018. Serum creatinine values reported in μmol/L were converted to mg/dL by dividing by 88.4.

**Table S3. Comprehensive clinical timeline for three affected family members (proband, father, elder sister)**

| **Time point** | **Proband** | **Father** | **Elder sister** |
| --- | --- | --- | --- |
| 2014  (9 years ago) | — | — | First‑pregnancy prenatal check‑up: elevated Scr/BUN; UA untested; no urate‑lowering therapy due to pregnancy contraindication |
| 2013  (12 years ago) | — | First detection of elevated Scr/BUN at age 42 y | — |
| Subsequent years | — | Progressive CKD | Underwent second pregnancy; urate‑lowering therapy deferred |
| 2023‑06 | Initial health‑check: elevated Scr, UA; eGFR 46 mL/min/1.73 m²; febuxostat 40 mg once‑daily prescribed, inconsistent adherence | Progressive CKD monitoring | — |
| 2024‑05 | Follow‑up visit | Routine renal monitoring | Confirmed hyperuricemia; initiated febuxostat 40 mg once‑daily |
| 2025‑06 | Regular follow‑up, ongoing febuxostat | Scr peaked at 1614 μmol/L; peritoneal dialysis initiated | Regular follow‑up, ongoing febuxostat |
| 2025‑10 | Genetic testing performed; UMOD c.274T>A heterozygous variant detected | Genetic testing performed; UMOD c.274T>A heterozygous variant detected | Genetic testing performed; UMOD c.274T>A heterozygous variant detected |

Footnote: y = years old; Scr = serum creatinine; BUN = blood urea nitrogen; UA = serum uric acid; CKD = chronic kidney disease.
